# Acute neuromuscular and metabolic responses to complex training with blood flow restriction: effects of arterial occlusion pressure on post-activation performance enhancement

**DOI:** 10.3389/fphys.2026.1932866

**Published:** 2026-09-29

**Authors:** Xiaohan Wang, Yin Yu, Jiayue Cui, Simin Lin, Qihao Sun, Bocheng Guo

**Affiliations:** 1 School of Sports Training, Wuhan Sports University, Wuhan, China; 2 Research Center for High-Quality Development of Characteristic Competitive Sports, Wuhan Sports University, Wuhan, China

**Keywords:** arterial occlusion pressure, blood flow restriction, complex training, countermovement jump, post-activation performance enhancement, surface electromyography

## Abstract

**Purpose:**

This study examined the acute effects of complex training (CT) with blood flow restriction (BFR) on countermovement jump (CMJ) performance, lower**-**limb muscle activation, and blood lactate (BLa) concentration in trained male collegiate athletes. It also aimed to identify the arterial occlusion pressure (AOP) level and recovery time window that produced the most favorable post**-**activation performance enhancement (PAPE) response.

**Methods:**

Using a randomized crossover design, twelve trained male collegiate athletes completed four experimental conditions: traditional CT and CT**-**BFR at 60%, 70%, and 80% AOP. CMJ performance (VJH: vertical jump height and RPP: relative peak power), muscle activation (RMS: root mean square and MF: median frequency), and BLa were assessed before the intervention and at 4, 8, 12, and 16 min following each protocol.

**Results:**

Traditional CT also induced significant changes in selected variables at specific recovery time points. However, the 70% AOP condition showed more consistent improvements among the tested conditions. Under 70% AOP, CMJ performance improved at 8 min, with significant increases in VJH (*p* < 0.05, ES = 0.37) and RPP (*p* < 0.05, ES = 0.94). RPP also remained elevated at 12 min (*p* < 0.05, ES = 0.77). This condition also showed more consistent sEMG responses, with increased RMS in the RF, VM, VL, and BF at 8 min, and changes in the VM and VL at 12 min (*p* < 0.05). MF changes were mainly observed in the VM and VL. In contrast, the 60% and 80% AOP conditions showed less consistent improvements in CMJ performance and muscle activation. BLa showed a significant time effect and AOP × time interaction, increasing markedly after the intervention, peaking at 4 min, and then progressively declining during recovery (*p* < 0.01).

**Conclusion:**

CT-BFR at 70% AOP showed a relatively favorable acute response among the tested conditions, especially for CMJ performance and thigh muscle activation during the 8–12 min recovery window. These findings provide preliminary evidence that 70% AOP may be a potentially suitable pressure level for CT**-**BFR based on the acute responses observed during PAPE based warm**-**up protocols.

## 1 Introduction

Post**-**activation performance enhancement (PAPE) refers to a transient improvement in muscular strength and power following high**-**intensity conditioning activities (CAs). This response is generally attributed to short**-**term changes in neurophysiological and contractile function, including myosin regulatory light**-**chain phosphorylation, increased motor unit recruitment, and greater motoneuron excitability (Hodgson et al., 2005; Tillin and Bishop, 2009). Other acute physiological responses, such as increased muscle temperature, changes in muscle water content, and enhanced muscle activation, may also contribute to improvements in voluntary strength and power after CAs (Cuenca-Fernández et al., 2017; Blazevich and Babault, 2019).

Previous studies have shown that PAPE is commonly induced by high**-**load resistance exercise, usually above 80% of one**-**repetition maximum (1RM) (Seitz and Haff, 2016; Xu et al., 2025). Although this approach can effectively stimulate the lower**-**limb neuromuscular system, it may also impose substantial mechanical stress and increase residual fatigue and injury risk. These factors may require longer recovery intervals and limit its use during periods of high training frequency or in season training. In addition, some traditional CAs (e.g., depth jumps and power cleans), may not fully match the movement patterns of explosive tasks such as jumping or sprinting. This mismatch may reduce the transfer of potentiation to subsequent sport**-**specific performance. Moreover, its practical application is limited because it requires the transportation of heavy training equipment to the training area. Therefore, alternative CA strategies are needed to induce acute performance enhancement while reducing mechanical strain and improving practical applicability.

Complex training (CT) has been proposed as an effective approach for improving muscular strength and power. It typically combines a high**-**load (HL) resistance exercise (80–90%1RM), followed by a plyometric training that has similar biomechanical characteristics (Robbins, 2005; Khamoui et al., 2009; Lesinski et al., 2016). Previous studies have shown that this strategy can improve the rate of force development and power output (Mitchell and Sale, 2011; Scott et al., 2017; Bauer et al., 2019). These effects may be partly explained by PAPE related mechanisms, which can contribute to increases in lower**-**limb power output and jump performance (Blagrove et al., 2019; Marshall et al., 2021). However, whether the neuromuscular stimulus induced by CT can be further enhanced by an additional metabolic stressor remains unclear.

Blood flow restriction (BFR) training has been used to increase neuromuscular and metabolic stress while keeping external loads relatively low (Centner et al., 2019; Benito et al., 2020; Hansen et al., 2020). Previous studies have shown that lower**-**limb BFR exercise may serve as an alternative to high**-**load resistance exercise because it can induce muscle and metabolic adaptations with less mechanical stress on the joints and soft tissues (Luebbers et al., 2019; Grønfeldt et al., 2020; Lorenz et al., 2021). In practice, BFR training is commonly performed with low loads (20–30% 1RM) and relatively high repetitions (15–30 repetitions per set) to provide sufficient metabolic stimulation (Pope et al., 2013; Patterson et al., 2019). Evidence from meta-analyses and experimental studies suggests that BFR may increase local hypoxia, metabolite accumulation, and muscle activation, which may contribute to acute neuromuscular responses during explosive exercise (Yamanaka et al., 2012; Yang et al., 2024).

Beyond its traditional application for chronic adaptations, recent studies have investigated whether BFR can induce PAPE. Doma et al. (2020) showed that a BFR**-**based CA induced PAPE and improved subsequent vertical jump performance, suggesting that BFR may serve as an alternative strategy for acute performance enhancement. Similarly, Cleary and Cook reported that BFR combined with CT elicited acute potentiation responses, indicating that BFR may enhance the acute effects of traditional CT (Cleary and Cook, 2020) A recent systematic review also suggested that BFR-based CAs have the potential to induce PAPE; however, the magnitude of these responses may depend on factors such as exercise mode, recovery interval, and BFR application parameters (Tian et al., 2022).

Because of its lower mechanical demand, BFR may also be useful when heavy external loading is not ideal. Recent studies further support this load-sparing advantage of BFR across different populations. Low-load BFR training has been shown to improve muscle thickness and functional outcomes in physically inactive adults (Ahmad et al., 2026), and BFR combined with exercise has improved muscle mass and lower-limb function in individuals with hemiplegia (Feng et al., 2025). Nevertheless, BFR training usually produces adaptations similar to those achieved with high**-**load resistance training, rather than clearly superior improvements (Manini and Clark, 2009; Yasuda et al., 2011; Pope et al., 2013). Therefore, combining BFR with CT may provide an additional metabolic stimulus to the neuromuscular stimulus induced by CT. However, whether this combination can further enhance acute PAPE responses remains unclear.

Although BFR has been widely studied in resistance exercise, its use within CT protocols remains less well understood (Cleary and Cook, 2020; Doma et al., 2020). Most previous studies on CT**-**BFR have focused on chronic adaptations, whereas evidence on its acute effects is still limited, especially for subsequent explosive performance. This is an important gap because many sports require athletes to produce high power within a short period. Examining the acute response to CT**-**BFR may therefore help clarify whether adding BFR can enhance the PAPE effect induced by CT.

Cuff pressure is another key issue in CT**-**BFR. A recent meta**-**analysis reported that many BFR studies did not provide a clear justification for the arterial occlusion pressure (AOP) used in their protocols (Clarkson et al., 2020). This is important because cuff pressure determines the degree of blood flow restriction and may influence the balance between metabolic stimulation and residual fatigue. Although individualized AOP based pressure settings are commonly recommended, the optimal pressure for inducing acute PAPE during CT**-**BFR remains unclear. Some empirical studies suggest that cuff pressure may affect training responses, with moderate pressures often showing favorable effects compared with very low (<50% AOP) or very high (≥80% AOP) pressure settings (Ilett et al., 2019; Das and Paton, 2022; Roehl et al., 2023). Based on this rationale, the present study selected three relative pressure levels, 60%, 70%, and 80% AOP, to examine whether different cuff pressures produce different acute responses in lower**-**limb explosive performance, muscle activation, and metabolic stress.

Therefore, the primary aim of this study was to examine whether CT**-**BFR performed at three AOP levels, 60%, 70%, and 80%, could induce acute PAPE. A further aim was to determine the time course of these responses at 4, 8, 12, and 16 min after the intervention in male collegiate athletes. Specifically, this study assessed changes in lower**-**limb explosive performance, muscle activation, and blood lactate (BLa) concentration under different cuff pressure and recovery**-**time conditions. These findings may contribute to a better understanding of acute CT-BFR responses under different cuff pressure conditions.

We hypothesized that CT**-**BFR would induce pressure dependent acute PAPE responses, with a moderate AOP level potentially providing a more favorable balance between metabolic stimulation and residual fatigue. This response was expected to be reflected by improved countermovement jump (CMJ) performance, greater lower**-**limb muscle activation, and a clear but not excessive BLa concentration response during recovery.

## 2 Materials and methods

### 2.1 Participants

Trained male collegiate athletes participated from volleyball and track and field participated in the study (n = 12; age: 20.7 ± 1.2 years; height: 180.2 ± 6.3 cm; weight: 72.48 ± 8.41 kg; absolute 1RM: 115.5 ± 12.5 kg; relative 1RM: 1.59 ± 0.23 kg·kg^-1^; thigh circumference: 58.3 ± 3.4 cm; sport-specific training experience: 7.8 ± 1.9 years; training frequency: 5 ± 1 sessions/week). Participants met the following inclusion criteria: ((1) held at least a National Level II Athlete qualification according to the Chinese athlete classification system, indicating systematic training and competitive experience, but not national-team or international elite status; (2) have engaged in regular systematic resistance training and plyometric training at least three per week for a minimum of one year; (3) no history of sports related injuries or illnesses within the previous six months, and no neurological or psychiatric disorders that could influence experimental outcomes. Assessment and implementation of the 1RM were conducted according to established strength and conditioning protocols (Warneke et al., 2023). Before the experiment, all participants completed a BFR related risk assessment questionnaire. During the sessions, RPE was recorded and no adverse events occurred.

All participants read and signed a free and informed consent form that contained all information relevant to the study. The study was conducted in accordance with the Declaration of Helsinki, and was approved by the Medical Ethics Committee of Wuhan Sports University (no. 2026045).

### 2.2 Experimental design

This study used a randomized, counterbalanced Latin square repeated-measures crossover design to examine the acute effects of CT-BFR on lower-limb explosive performance in trained male collegiate athletes. Participants were randomly allocated to four sequence groups (n = 3 per group) according to a balanced Latin square design. Each participant completed four experimental conditions: traditional CT without cuff application and CT**-**BFR at 60%, 70%, and 80% of AOP. Before the experimental sessions, each participant completed a 1RM back squat test on a separate testing day. This test was used to determine the individual training load. Participants also completed maximal voluntary contraction (MVC) tests so that the surface electromyography (sEMG) root mean square (RMS) signals could be normalized. In addition, they were familiarized with all testing procedures. These preliminary sessions were separated by 72 h (± 0.5 h).

Each participant then completed four experimental sessions in separate weeks. These included one traditional CT condition without cuff application and three CT**-**BFR conditions at 60%, 70%, and 80% AOP. A washout period of at least 7 days was used between sessions to reduce residual fatigue and carryover effects. To limit the influence of circadian variation, all sessions were performed at the same time of day. Participants were also instructed to avoid caffeine and strenuous exercise for at least 48 h before each session. All tests were conducted in a laboratory environment maintained at approximately 24 °C. Jump performance was assessed using the CMJ. Acute PAPE responses were determined by comparing jump performance before and after each intervention. A PRE CMJ assessment was performed before each experimental session.

The cuffs were applied only during the intervention protocol and were not used during the warm-up or baseline CMJ assessment. During the CT**-**BFR conditions, participants performed the squat and half squat jump while wearing pneumatic cuffs connected to a portable intelligent pressurization device (KAATSU SMART; Yijia Yuan Sports Technology Development Co., Ltd., Beijing, China). The cuffs were placed at the most proximal portion of both thighs. The cuff width was 5 cm. Cuff pressure was continuously monitored and maintained by the device throughout the intervention. Cuff pressures were assigned according to thigh circumference with reference to the methods reported by Loenneke et al. and Wei et al (Loenneke et al., 2015; Wei et al., 2021). The final pressure settings were adapted to the 10 mmHg adjustment increments permitted by the device, and the actual pressures applied are presented in **Table 1**. In the control condition, participants completed the same CT protocol without cuff application. The exercise protocol, including exercise mode, sets, repetitions, and load, was based on previous CT and CT**-**BFR studies (Lixandrão et al., 2018; Cleary and Cook, 2020; Grønfeldt et al., 2020; Liu et al., 2022; Zhou et al., 2024). The detailed protocol is shown in **Table 2**.

**Table 1:** BFR pressures (Loenneke et al., 2015; Wei et al., 2021).

Thigh circumference (cm)	Pressure used (mmHg)		
	60%AOP	70%AOP	80%AOP
<45-50.9	120	140	160
51-55.9	150	180	210
56-59.9	180	210	240
≥60	210	250	290

PRE, baseline measurement before the conditioning activity; AOP, arterial occlusion pressure; CT, complex training; BFR, blood flow restriction; CMJ, countermovement jump; sEMG, surface electromyography; BLa, blood lactate concentration.

**Table 2:** Experimental conditioning protocols.

Condition	Back squat (sets × reps × load)	Half-squat jump (sets × reps × load)	Rest between exercises	Rest between sets	Cuff pressure	Cuff state
Traditional CT	2 × 2 × 85% 1RM	2 × 10 × 30% 1RM	30 s	3 min	No cuff	No cuff
CT-BFR 60AOP	2 × 20 × 30% 1RM	2 × 10 × 30% 1RM	30 s	3 min	60%AOP	Continuous
CT-BFR 70AOP	2 × 20 × 30% 1RM	2 × 10 × 30% 1RM	30 s	3 min	70%AOP	Continuous
CT-BFR 80AOP	2 × 20 × 30% 1RM	2 × 10 × 30% 1RM	30 s	3 min	80%AOP	Continuous

CT, complex training; BFR, blood flow restriction; AOP, arterial occlusion pressure; 1RM, one-repetition maximum. In the CT-BFR conditions, cuff pressure was applied continuously throughout the intervention. Absolute cuff pressures were according to thigh circumference, as shown in **Table 1**.

### 2.3 Testing procedures

On each testing day, participants arrived at the laboratory at a predetermined time and remained seated for 10 min. A trained researcher collected approximately 0.8 μL of fresh fingertip capillary blood from the middle finger of the right hand at each measurement point. BLa concentration was determined using a portable lactate meter (Eaglenos Sciences, Inc., Nanjing, China; manufacturer-reported precision CV ≤ 6%). After a further 10 min of rest, participants completed a standardized 15 min warm**-**up before formal testing. The warm**-**up included 5 min of myofascial release, 5 min of light jogging, and 5 min of dynamic stretching (Carvalho et al., 2012). After the warm-up, participants rested for 5 min before the PRE CMJ assessments. Three CMJ trials were performed at PRE and at each post-intervention time point (4, 8, 12, and 16 min), with 15 s of rest between attempts (Barreto et al., 2023). Each CMJ assessment block lasted approximately 50–60 s depending on individual execution time, and the reported recovery time points indicated the start of each CMJ assessment rather than the exact completion time of all trials. After the PRE CMJ assessment, participants rested for 3 min before initiating the intervention protocol.

After the intervention, CMJ performance was assessed at 4, 8, 12, and 16 min. These time points were selected based on previous PAPE studies indicating that performance enhancement may emerge after several minutes of recovery following a CA (Mitchell and Sale, 2011). Recent studies using BFR as a CA have also reported improved explosive performance within this recovery period (Cleary and Cook, 2020; Doma et al., 2020). At each recovery time point, BLa was measured first, followed by three CMJ trials. During each CMJ trial, sEMG signals were recorded from the lower**-**limb muscles (**Figure 1**).

**Figure 1:**
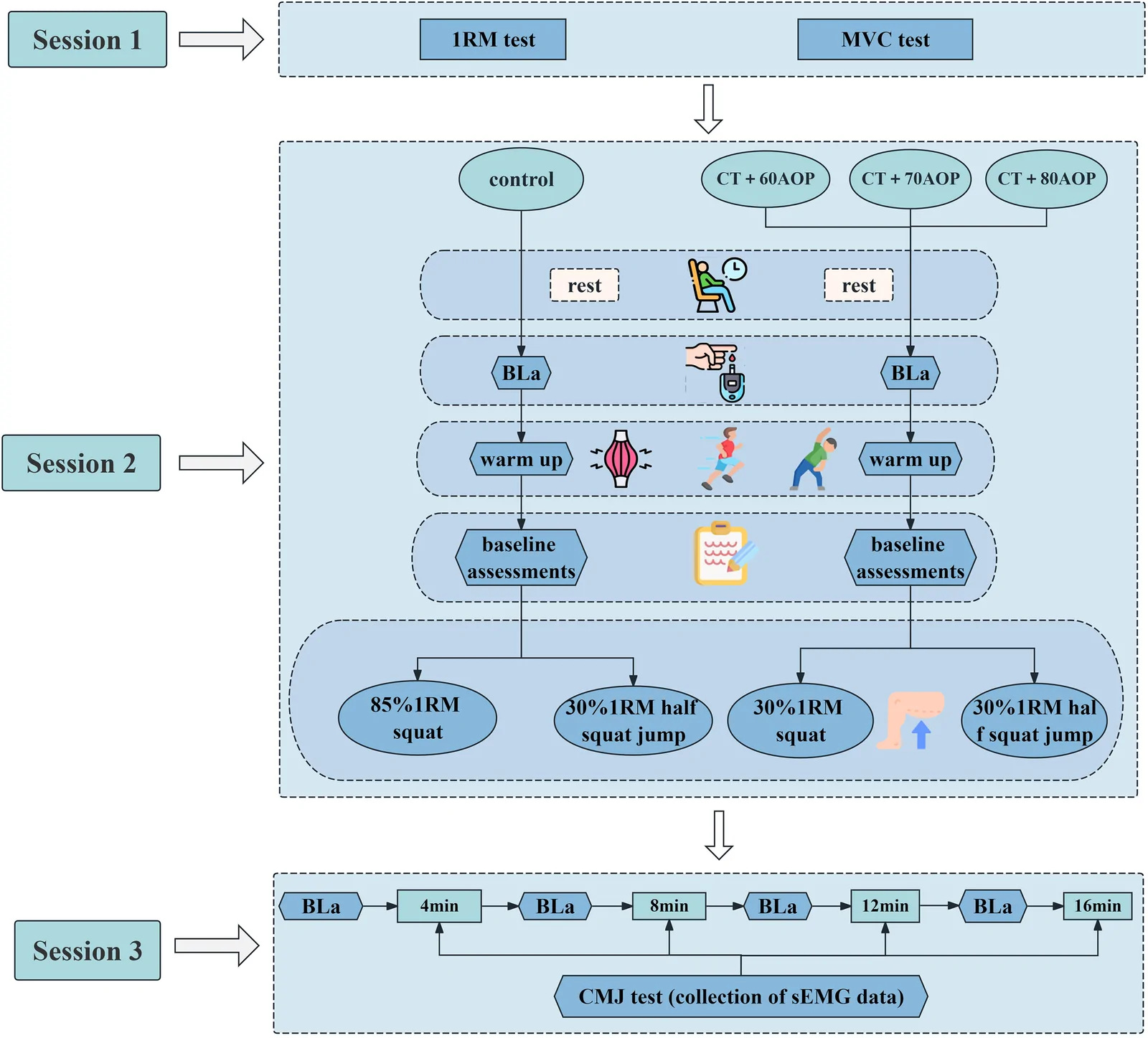
Experimental design and testing procedures. PRE, baseline measurement before the conditioning activity; AOP, arterial occlusion pressure; CT, complex training; BFR, blood flow restriction; CMJ, countermovement jump; sEMG, surface electromyography; BLa, blood lactate concentration.

### 2.4 Measurements

#### 2.4.1 1RM test

The 1RM back squat test was performed according to the National Strength and Conditioning Association (NSCA) guidelines. Participants stood with their feet approximately shoulder width apart, with the toes slightly turned outward. They descended until the thighs were parallel to the floor and then returned to the starting position by extending the hips and knees. At least two trained investigators supervised each test to ensure participant safety. Before testing, participants completed a progressive warm**-**up consisting of 8–10 repetitions with a light load, followed by 3–5 repetitions after a 10%–20% increase in load. After a further increase, participants completed 2–3 repetitions before attempting the 1RM lift. Rest intervals of 1–4 min were provided between sets according to the testing stage. If the lift was successful, the load was increased for the next attempt. If unsuccessful, the load was reduced by 5%–10%. The highest load completed with proper technique was recorded as the participant’s 1RM. The 1RM was typically determined within three to five attempts (Haff and Triplett, 2016).

#### 2.4.2 CMJ test

The CMJ test is a reliable method for assessing lower**-**limb power output, including vertical jump height (VJH) and relative peak power (RPP). During the CMJ test, participants kept their hands on their hips to eliminate the influence of arm swing. For the CMJ protocol, participants stood with their feet shoulder width apart. After an auditory cue, they were instructed to perform a rapid downward movement and flex the knee joint to approximately 90°. They then immediately executed a maximal vertical jump with a stable landing (Markovic et al., 2004; McMahon et al., 2018). During testing, participants performed three CMJ tests on a force platform (Kistler 9260AA6, KISTLER, Switzerland, 1200 Hz). The trial with the highest jump height was selected, and the corresponding CMJ performance variables (including VJH and RPP) and sEMG parameters obtained from that trial were used for subsequent analysis. CMJ performance and lower**-**limb muscle sEMG activity were recorded during each trial. The reliability of CMJ variables was acceptable (ICC(3,1) = 0.903 and CV = 3.21% for VJH; ICC(3,1) = 0.871 and CV = 4.95% for RPP).

#### 2.4.3 Collection and processing of surface EMG data

##### 2.4.3.1 Electrode placement and requirements

sEMG electrodes were placed on six major muscle conditions of the dominant leg that are highly involved in jumping: rectus femoris (RF), vastus medialis (VM), vastus lateralis (VL), biceps femoris (BF), tibialis anterior (TA), and medial gastrocnemius (MG) (Mrdakovic et al., 2008; Padulo et al., 2013). sEMG signals were collected using a 16 channel wireless system (Noraxon, USA, 2000 Hz). Bipolar Ag/AgCl electrodes were placed parallel to the muscle fiber direction and positioned at the midpoint between the muscle origin and insertion. The inter electrode distance was maintained at 20 mm. Electrode placement followed the SENIAM recommendations (www.seniam.org). Before electrode placement, the skin at each site was shaved, lightly abraded, and cleaned with ethanol to reduce skin impedance.

##### 2.4.3.2 MVC test and RMS value collection

After electrode placement, the electrode wires were connected to record MVC of the thigh muscles, and the corresponding sEMG signals were collected. To normalize EMG activity, MVC tests were performed for each muscle condition before the exercise trials. The MVC procedures were performed according to commonly used surface electromyography normalization protocols (Hermens et al., 2000). Each MVC trial was performed for approximately 8 s in standardized manual muscle testing positions reflecting the primary function of each muscle. A 1 min rest interval was provided between trials. Participants were instructed to reach maximal force as quickly as possible and to maintain maximal effort throughout each contraction. Standardized verbal encouragement was provided by the same researcher during all MVC trials. Two MVC trials were recorded for each muscle, and the RMS signal from the middle 3 s (excluding the first and last second) of each trial was averaged and used as the normalization reference. The RMS value obtained during each test was divided by the corresponding EMG amplitude recorded during MVC to normalize muscle activation. The normalized EMG activity during post intervention testing was expressed as a percentage of MVC (%MVC), calculated as: %MVC = (RMS during muscle activity/RMS during MVC) × 100.

##### 2.4.3.3 sEMG data collection and processing

Raw sEMG signals were processed using the manufacturer’s proprietary software (myoRESEARCH MR3, version 3.20.68, Noraxon, USA). The system provides low baseline noise (< 2 μV) and high input resolution (24 bit). EMG data were recorded synchronously with force signals from the Kistler force platform system. Signal processing included digital filtering, rectification, smoothing, and amplitude normalization. Raw EMG signals were processed offline in MR3 software using a fourth order Butterworth band pass filter (20–450 Hz). The signals were then rectified and smoothed. Baseline noise and signal fidelity were maintained within manufacturer specifications (< 2 μV). Finally, RMS and median frequency (MF) were extracted for further analysis.

### 2.5 Statistical analysis

All statistical analyses were performed using IBM SPSS Statistics (version 26.0, IBM, Chicago, IL, United States). Data are presented as the mean ± standard deviation (SD). The normality of the data distribution was assessed using the Shapiro–Wilk test. A two way repeated measures analysis of variance (ANOVA) was used to examine the main effects of condition (0%, 60%, 70%, and 80% AOP), time (PRE, 4, 8, 12, and 16 min), and their interaction on each dependent variable. Mauchly’s test was used to assess the assumption of sphericity. When this assumption was violated, the Greenhouse–Geisser correction was applied. Bonferroni adjusted *post hoc* tests were performed when significant main or interaction effects were observed. Cohen’s d effect sizes (ES) as follows: <0.2, trivial; 0.2–0.6, small; 0.6–1.2, moderate; and >1.2, large. Statistical significance was set at *p* < 0.05. CMJ performance variables were considered the primary outcomes, whereas sEMG variables and BLa were considered secondary physiological outcomes.

## 3 Results

The outcome measures were used to evaluate the acute effects of each experimental condition across time on: (1) CMJ performance (VJH and RPP); (2) muscle activation, represented by the RMS of EMG signals; (3) neuromuscular function and fatigue, as indicated by the MF of the EMG spectrum; (4) metabolic fatigue, reflected by the BLa.

### 3.1 CMJ

CMJ performance under the four experimental conditions was analyzed using a two**-**way repeated**-**measures ANOVA (condition × time). For VJH, significant main effects of condition (F(1.99,21.84) = 24.551, *p* < 0.001, ηp² = 0.691) and time (F(2.37,26.03) = 17.847, *p* < 0.001, ηp² = 0.619) were observed. A significant condition × time interaction was also found (F(3.76,41.35) = 4.111, *p* < 0.05, ηp² = 0.272). For RPP, the main effect of condition was not significant (F(2,05,22.59) = 1.267, *p* = 0.302, ηp² = 0.103). However, a significant main effect of time was observed (F(2.57,28.29) = 37.680, *p* < 0.001, ηp² = 0.774), together with a significant condition × time interaction (F(4.38,48.15) = 8.536, *p* < 0.001, ηp² = 0.437). *Post hoc* analyses showed that VJH and RPP increased significantly at 8 min after the intervention in the CT and 70% AOP conditions. In the 70% AOP condition, RPP also remained significantly higher at 12 min. No significant changes were observed at any time point in the 60% or 80% AOP conditions (**Figure 2**; **Table 3**).

**Figure 2:**
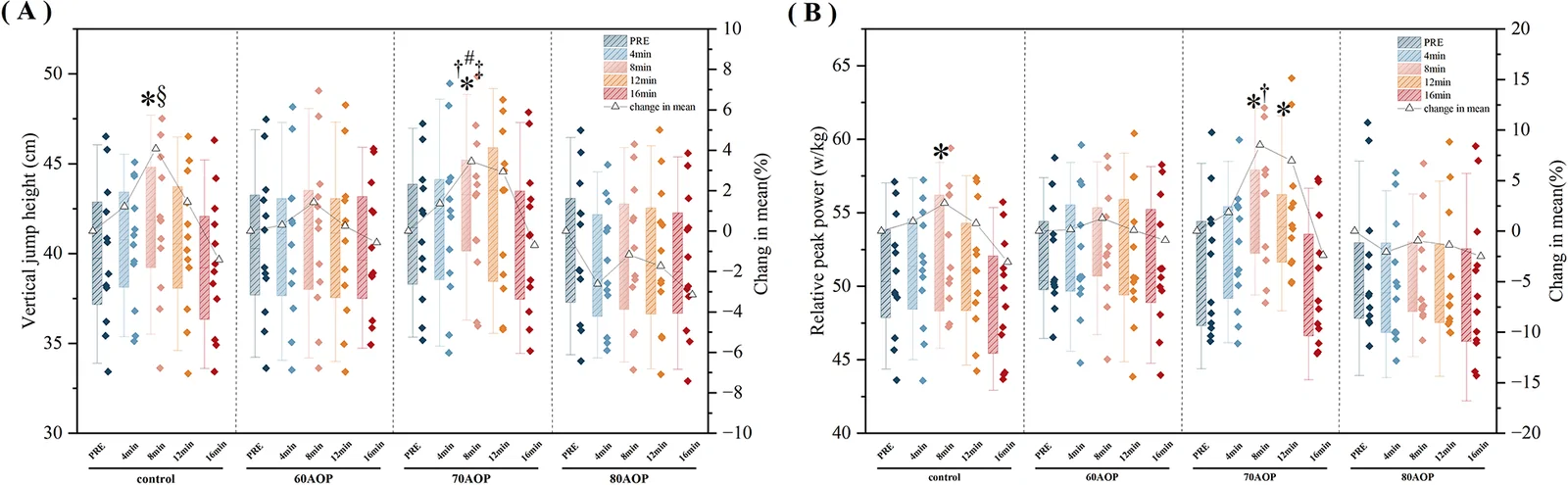
Change in CMJ performance between 0, 60, 70 and 80 conditions across time points [**(A)** Vertical jump height; **(B)** Relative peak power]. * Significantly different from PRE. Statistical significance was set at *p* < 0.05. AOP, Arterial Occlusion Pressure; VJH, vertical jump height; RPP, relative peak power. ^#^60AOP compared with 70AOP at the same time point (*p* < 0.05). ^†^70AOP compared with 80AOP at the same time point (*p* < 0.05). ^‡^control compared with 70AOP at the same time point (*p* < 0.05). ^§^control compared with 80AOP at the same time point (*p* < 0.05).

**Table 3:** Change in CMJ performance variables between 0, 60, 70 and 80 conditions across time points.

Condition	Time	VJH				RPP			
		Mean ± SD (cm)	Change (%)	ES	Mean difference (95% CI)	Mean ± SD (w/kg)	Change (%)	ES	Mean difference (95% CI)
control	PRE	39.98 ± 4.05	\	\	\	50.71 ± 4.22	\	\	\
	4min	40.46 ± 3.39	1.21	0.12	(-1.658,0.694)	51.19 ± 4.13	0.95	0.11	(-1.458,0.498)
	8min	41.60 ± 4.06*	4.07	0.40	(-3.017,-0.235)	52.10 ± 4.22*	2.75	0.33	(-1.599,-0.322)
	12min	40.55 ± 3.96	1.43	0.14	(-1.282,0.135)	51.09 ± 4.29	0.76	0.10	(-1.534,0.767)
	16min	39.41 ± 3.87	-1.42	-0.14	(-0.154,1.288)	49.14 ± 4.15	-3.08	-0.37	(-0.481,2.646)
60AOP	PRE	40.56 ± 4.22	\	\	\	51.92 ± 3.64	\	\	\
	4min	40.68 ± 4.41	0.3	0.03	(-0.747,0.505)	52.01 ± 4.28	0.16	0.02	(-0.979,-0.813)
	8min	41.14 ± 4.63	1.42	0.14	(-1.533,0.379)	52.59 ± 3.92	1.28	0.18	(-2.219,0.800)
	12min	40.66 ± 4.45	0.25	0.02	(-0.813, 0.610)	51.97 ± 4.73	0.08	0.01	(-1.361,1.278)
	16min	40.33 ± 3.72	-0.57	-0.06	(-0.559,1.021)	51.45 ± 4.46	-0.92	-0.13	(-1.182,2.138)
70AOP	PRE	41.16 ± 3.88	\	\	\	51.38 ± 4.66	\	\	\
	4min	41.72 ± 4.58	1.36	0.14	(-1.584,0.464)	52.33 ± 4.11	1.85	0.20	(-3.867,1.967)
	8min	42.58 ± 4.18*	3.44	0.37	(-2.784,-0.052)	55.75 ± 4.24*	8.51	0.94	(-7.887,-0.856)
	12min	42.37 ± 4.54	2.93	0.31	(-2.459,0.043)	54.96 ± 4.41*	6.96	0.77	(-6.946,-0.204)
	16min	40.87 ± 4.29	-0.70	-0.08	(-0.655,1.234)	50.15 ± 4.35	-2.39	-0.26	(-0.462,2.922)
80AOP	PRE	40.41 ± 4.03	\	\	\	51.22 ± 4.86	\	\	\
	4min	39.35 ± 3.47	-2.61	-0.26	(0.005,2.075)	50.15 ± 4.24	-2.09	-0.22	(-0.262,2.407)
	8min	39.94 ± 3.98	-1.17	-0.12	(0.168,0.781)	50.74 ± 3.69	-0.95	-0.10	(-0.901,1.870)
	12min	39.71 ± 4.24	-1.73	-0.17	(0.277,0.953)	50.52 ± 4.42	-1.38	-0.15	(-0.460,1.876)
	16min	39.14 ± 4.47	-3.15	-0.32	(0.522,1.354)	49.95 ± 5.16	-2.5	-0.26	(0.086,2.472)
ANOVA results (P value, F value, ηp2)	condition	F(3,33) = 24.511, p < 0.001, ηp2 = 0.691				F(3,33) = 1.267, p = 0.302, ηp2 = 0.103			
	time	F(2.366,26.029) = 17.847, p < 0.001, ηp2 = 0.619				F(2.572,28.291) = 37.680, p < 0.001, ηp2 = 0.774			
	condition × time	F(3.759,41.353) = 4.111, p < 0.05, ηp2 = 0.272				F(4.377,48.146) = 8.536, p < 0.001, ηp2 = 0.437			

*Significantly different from PRE. Values are presented as mean ± SD [Mean difference (95% CI)]. VJH, vertical jump height; RPP, relative peak power. Statistical significance was set at *p* < 0.05.

### 3.2 RMS and MF

Changes in RMS and MF under the four experimental conditions were analyzed using a two**-**way repeated**-**measures ANOVA (condition × time). For RMS, significant main effects of condition were observed for VM (F(2.61,28.70) = 3.744, *p* < 0.05, ηp² = 0.254) and VL (F(2.33,25.65) = 3.661, *p* < 0.05, ηp² = 0.250). Significant main effects of time were found for RF (F(2.60,28.63) = 20.024, *p* < 0.001, ηp² = 0.645), VM(F(2.67,29.41) = 4.324, *p* < 0.05, ηp² = 0.282), VL (F(3.68,40.47) = 6.265, *p* < 0.001, ηp² = 0.363), and BF (F(2.96,32.51) = 11.561, *p* < 0.001, ηp² = 0.512). Significant condition × time interactions were also observed for VM (F(4.65,51.16) = 3.309, *p* < 0.05, ηp² = 0.231) and VL (F(5.20,57.22) = 5.089, *p* < 0.001, ηp² = 0.316) (**Figure 3**). For MF, significant main effects of condition were found for RF (F(2.59,28.47) = 18.504, *p* < 0.001, ηp² = 0.627), VM (F(2.61,28.69) = 32.521, *p* < 0.001, ηp² = 0.747), and VL (F(2.68,29.51) = 22.262, *p* < 0.001, ηp² = 0.669). Significant main effects of time were observed for VM (F(2.61,28.76) = 6.172, *p* < 0.05, ηp² = 0.359) and VL (F(2.86,31.51) = 5.842, *p* < 0.05, ηp² = 0.347) (**Figure 4**).

**Figure 3:**
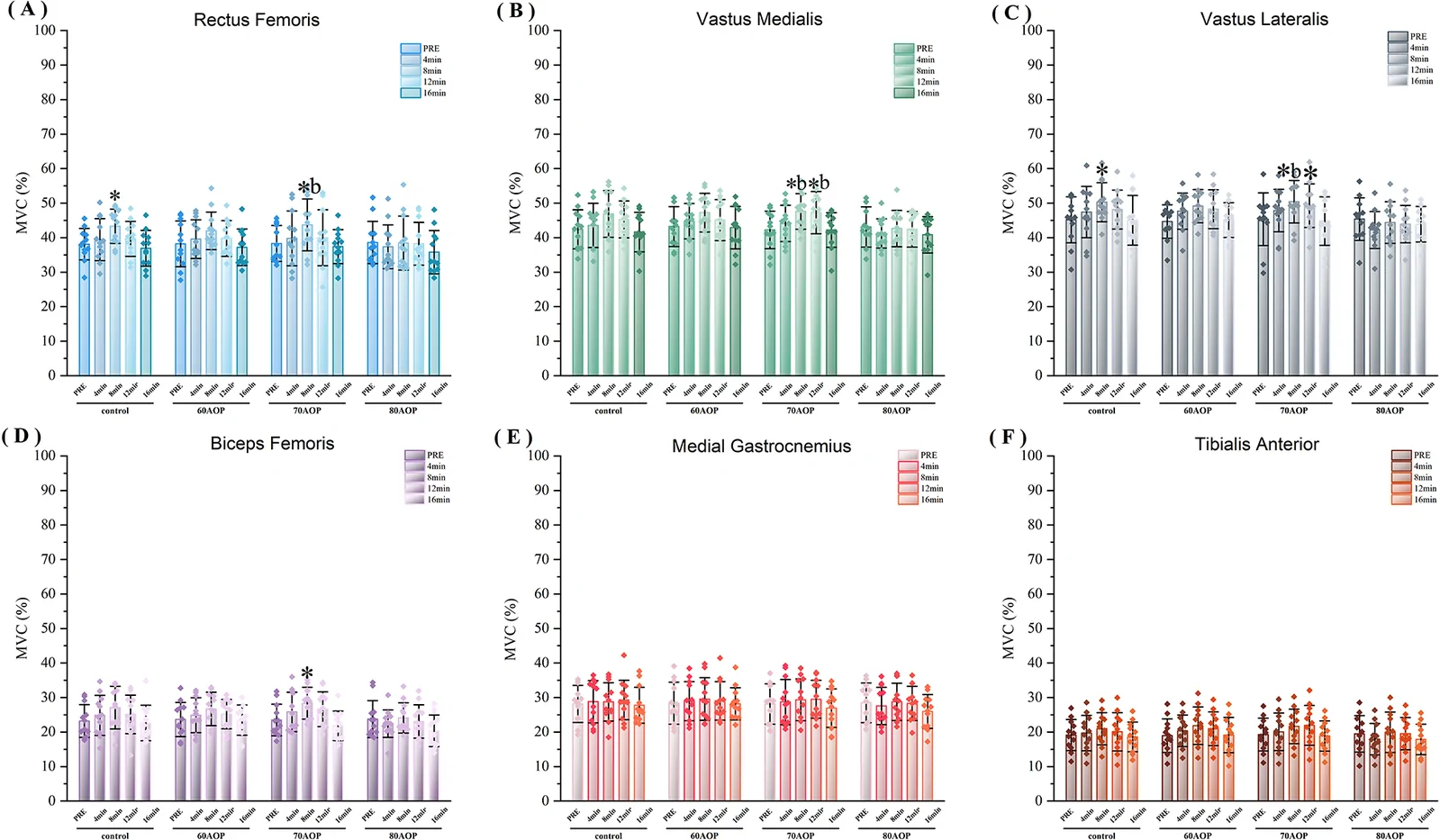
Change in RMS values between 0, 60, 70 and 80 conditions across time points (**(A)** Rectus Femoris; **(B)** Vastus Medialis; **(C)** Vastus Lateralis; **(D)** Biceps Femoris; **(E)** Medial Gastrocnemius; **(F)** Tibialis Anterior). *Significantly different from PRE. Statistical significance was set at *p* < 0.05. AOP, Arterial Occlusion Pressure. a: 60AOP compared with 70AOP at the same time point (*p* < 0.05). b: 70AOP compared with 80AOP at the same time point (*p* < 0.05).

**Figure 4:**
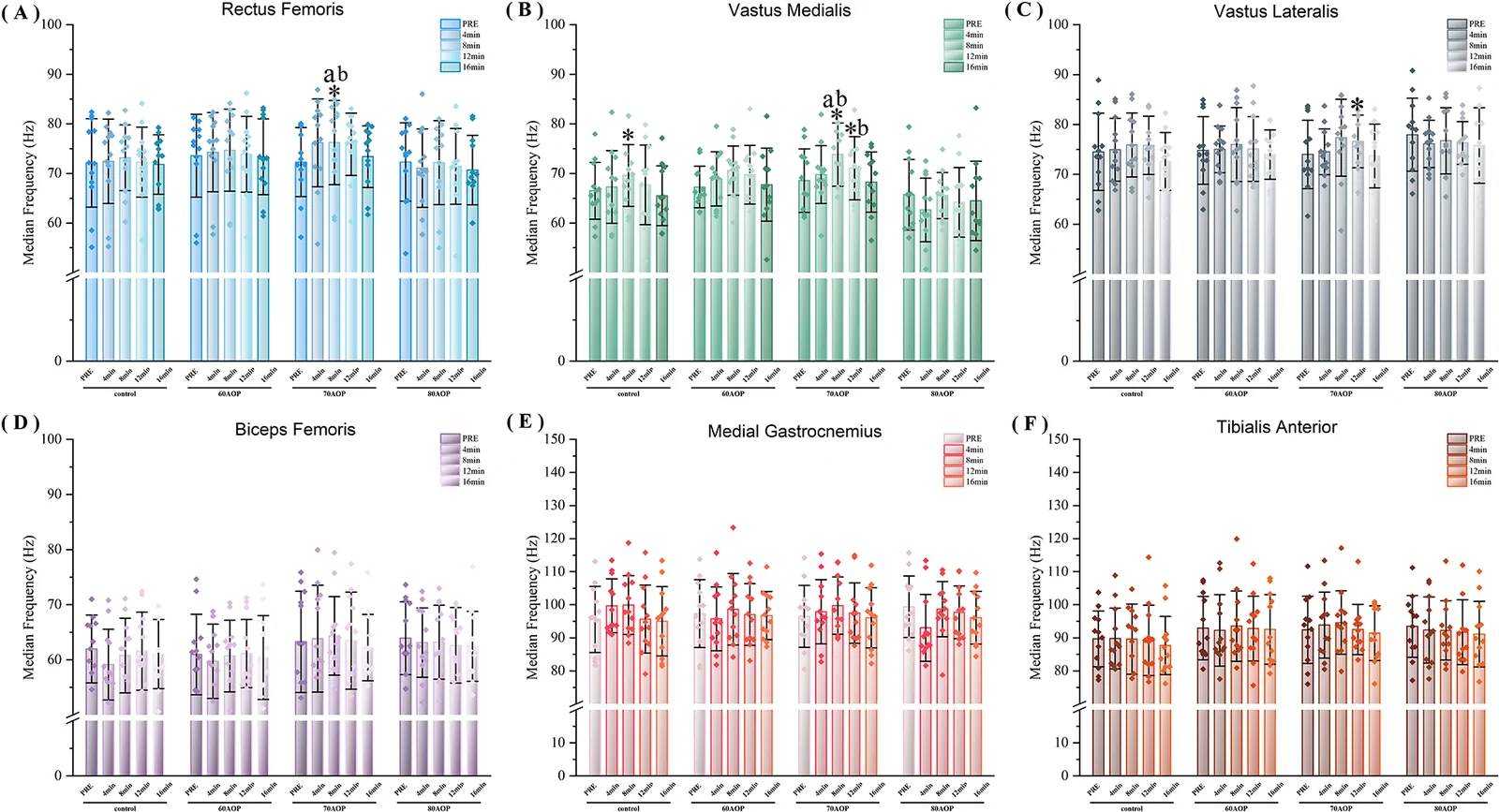
Change in MF values between 0, 60, 70 and 80 conditions across time points [**(A)** Rectus Femoris; **(B)** Vastus Medialis; **(C)** Vastus Lateralis; **(D)** Biceps Femoris; **(E)** Medial Gastrocnemius; **(F)** Tibialis Anterior]. *Significantly different from PRE. Statistical significance was set at *p* < 0.05. AOP, Arterial Occlusion Pressure. a: 60AOP compared with 70AOP at the same time point (*p* < 0.05). b: 70AOP compared with 80AOP at the same time point (*p* < 0.05).

### 3.3 BLa concentration

BLa concentration at each time point (PRE, 4, 8, 12, and 16 min) under the different AOP conditions was analyzed using a two**-**way repeated**-**measures ANOVA (AOP × time). BLa concentrations before and after the conditioning activity are presented in **Figure 5**. There was a significant main effect of time, with BLa increasing sharply after the intervention, peaking at 4 min, and then gradually decreasing at 8, 12, and 16 min (F(1.63,17.90) = 432.295, *p* < 0.001, ηp² = 0.975). A significant condition × time interaction was also observed for BLa (F(4.55,50.03) = 3.318, *p* < 0.001, ηp² = 0.232). Specifically, the 70% and 80% AOP conditions showed higher BLa responses at 4 and 8 min, whereas the 60% AOP condition maintained relatively higher BLa values at 12 min. By 16 min, BLa values had declined toward PRE levels in all conditions, and the differences between AOP conditions became smaller (**Figure 5**).

**Figure 5:**
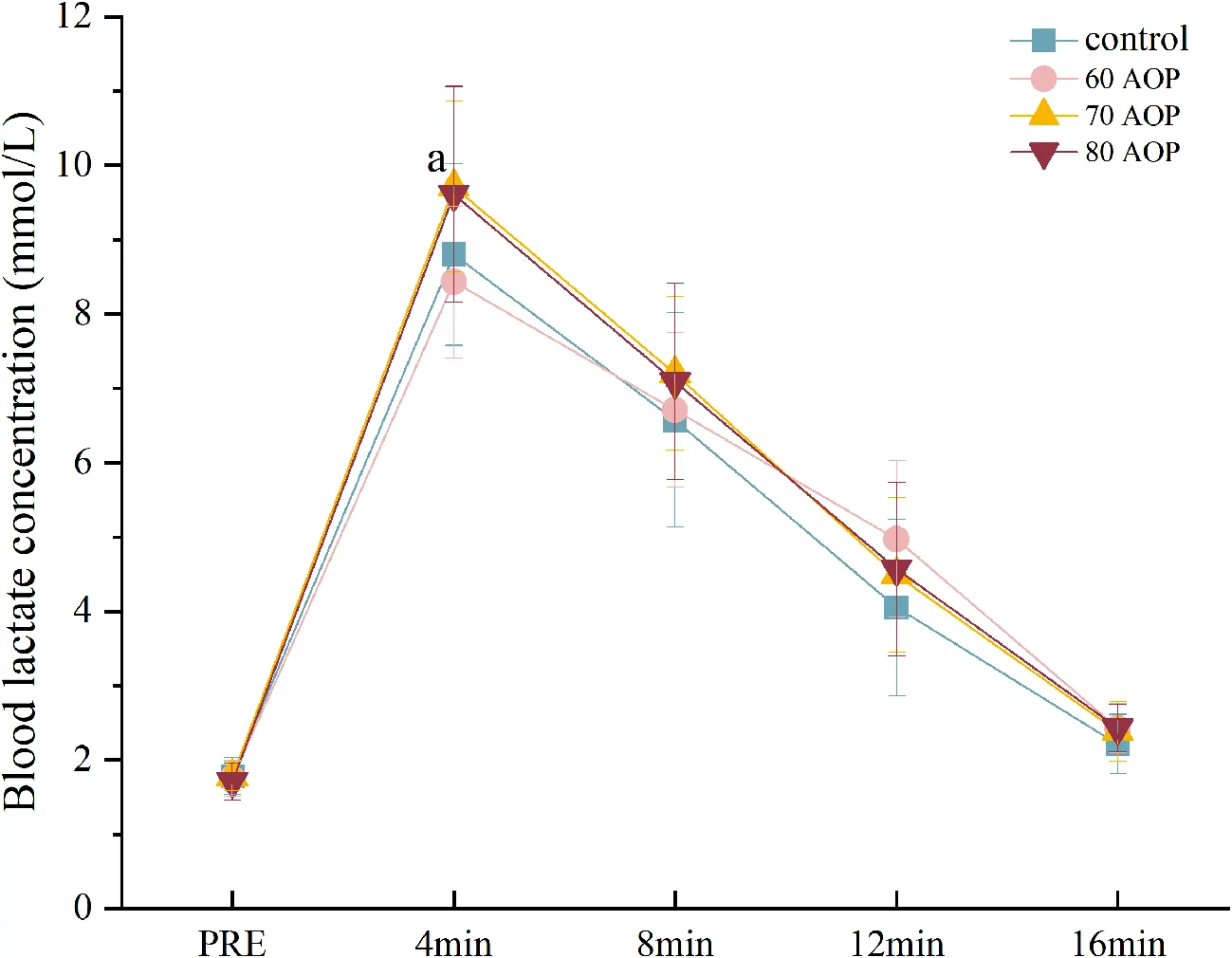
Changes in BLa concentration and comparison between 0, 60, 70 and 80 conditions across time points. AOP, Arterial Occlusion Pressure. Data are presented as mean ± SD. *Indicates significant difference from PRE at *p* < 0.01. a: 60AOP compared with 70AOP at the same time point (*p* < 0.05).

## 4 Discussion

The present study compared the acute PAPE responses induced by CT alone and CT**-**BFR under different cuff pressure conditions. The main finding was that the 70% AOP condition showed a relatively favorable acute response pattern among the tested conditions. Compared with 60% and 80% AOP, 70% AOP produced clearer improvements in CMJ performance. The sEMG and BLa results also showed a more suitable balance between muscle activation and metabolic stress under this pressure. In contrast, 60% AOP may have provided a weak stimulus, whereas 80% AOP seemed to produce more fatigue. These findings suggest that the PAPE response to CT**-**BFR may be influenced by cuff pressure, and that 70% AOP may provide a better balance between stimulation and fatigue.

Previous studies have shown that the improvement in jump performance after CT may be partly explained by the mechanical and neuromuscular characteristics of the exercise sequence (Kubo et al., 2007; Battaglia et al., 2014). In the present study, the CT protocol included the squat and the half**-**squat jump. Both exercises are multi**-**joint lower**-**limb movements involving the hip, knee, and ankle. The squat provided a loaded mechanical stimulus, whereas the half**-**squat jump provided a rapid eccentric**-**to**-**concentric explosive stimulus (Loturco et al., 2023; Thompson et al., 2023). This combination may have prepared the neuromuscular system for the following CMJ task. It may also explain why RPP improved at 8 and 12 min, while VJH increased mainly at 8 min under 70% AOP.

The time course of PAPE is usually explained by the balance between potentiation and fatigue. However, performance improves only when the potentiation effect is greater than the remaining fatigue. When fatigue is still dominant, performance enhancement may be reduced or delayed (Seitz and Haff, 2016; Li et al., 2024). Recovery time is therefore important. Previous evidence suggests that PAPE commonly occurs within 0–15 min after CAs, but the optimal recovery interval remains debated. Very short intervals (0–3 min) may be limited by fatigue, whereas moderate intervals (7–12 min) may allow potentiation to become more evident (Gouvea et al., 2013; Wilson et al., 2013; Chen et al., 2023). However, this recovery window should not be considered universal. A recent meta-analysis on BFR induced PAP responses further indicated that acute performance responses may vary according to exercise characteristics, cuff pressure, and testing protocols, suggesting that the optimal recovery interval may depend on the specific BFR application (Wang et al., 2023).

In the present study, CMJ performance improved mainly at 8 and 12 min under the 70% AOP condition. The timing of PAPE responses following BFR conditioning may differ across studies. Zheng et al. reported reduced CMJ performance during the early recovery phase, with improvement observed only after 12 min of recovery (Zheng et al., 2025). The different response patterns may be related to variations in BFR pressure, exercise volume, and conditioning activity characteristics, which may influence the balance between fatigue and potentiation. Therefore, the 8–12 min recovery window observed in this study should not be considered universal. The optimal recovery interval may instead depend on factors such as BFR pressure, exercise volume, CA characteristics, and individual differences (Gouvea et al., 2013; Wilson et al., 2013; Seitz and Haff, 2016).

The improvement in RPP under 70% AOP suggests that CT**-**BFR improved the ability to produce force rapidly after a short recovery period. However, the CMJ variables did not change in the same way. RPP improved at 8 and 12 min, whereas VJH showed a clear improvement mainly at 8 min. This means that CT**-**BFR may have improved instantaneous power output, but this effect was not fully transferred to overall jump height. RPP may be more sensitive to acute neuromuscular changes, whereas VJH is a more integrated performance outcome influenced by total impulse, movement coordination, take**-**off mechanics, and individual jump strategy. Therefore, the performance response should not be attributed to BFR alone. The biomechanical similarity between the CA and the subsequent CMJ task should also be considered, as previous studies have shown that task similarity may enhance the PAPE response (Doma et al., 2016; Kennedy and Drake, 2017).

Adding BFR to CT exposed the working muscles to repeated contractions and external cuff pressure at the same time. This condition can reduce arterial inflow to some extent and restrict venous return. As a result, local hypoxia and metabolite accumulation may increase (Patterson et al., 2019; Pignanelli et al., 2021). In this environment, slow**-**twitch fibers may fatigue earlier, and greater neural drive may be needed to maintain force output. This can increase the contribution of higher**-**threshold motor units, which usually innervate type II muscle fibers and are important for explosive force production (Pearson and Hussain, 2015; Davies et al., 2016). A similar acute performance response was reported by Wilk et al. (2022), who found that short**-**term BFR increased peak power (ES = 1.67), mean power (ES = 0.93), peak bar velocity (ES = 1.79), and mean bar velocity (ES = 1.36) during the bench press. Although their study used an upper**-**body task, the findings support the idea that BFR may acutely enhance mechanical power output under certain exercise conditions. More relevant lower**-**limb evidence was reported by Doma et al. (2020), who found that lunge exercise with BFR improved jump height (~4.5% ± 0.8%) and power output (~4.1% ± 0.3%) within 6–15 min after exercise. These findings are consistent with the present results and suggest that suitable BFR may enhance the acute neuromuscular stimulus during CT.

The sEMG results further support the neuromuscular interpretation. RMS is commonly used to reflect the amplitude of muscle activation during a specific movement task. In the present study, several thigh muscles showed increased normalized RMS values after the intervention, especially under 70% AOP. This suggests that the neuromuscular system may have increased muscle activation during the subsequent CMJ to meet the demand for rapid force production. The quadriceps muscles, including RF, VM, and VL, are important for knee extension during the propulsive phase of the CMJ. Greater activation of these muscles may therefore contribute to higher power output during take**-**off. The BF may also contribute through hip extension, knee joint control, and lower**-**limb stability. A previous meta**-**analysis showed that BFRT significantly enhanced RMS values of lower**-**limb muscles (SMD: 0.98; 95% CI: 0.71 to 1.224) (Wang et al., 2023). The present findings are generally consistent with this evidence. These muscle specific responses may help explain why 70% AOP produced more favorable CMJ related outcomes.

The cuff position may also help explain the muscle**-**specific EMG responses. In this study, the cuff was applied proximally on the thigh. Thus, the thigh muscles may have received a more direct restriction**-**related stimulus than the lower**-**leg muscles. This may explain why RF, VM, VL, and BF showed clearer changes than distal muscles (MG and TA). However, this interpretation should be cautious because sEMG during CMJ is affected by strategy, coordination, fatigue, and individual differences. Increased RMS therefore reflects greater task**-**related muscle activation, not direct evidence of neural potentiation. Moreover, the present findings are not fully consistent with those of Cleary and Cook (2020), who observed lower EMG amplitudes of the VL and BF during low**-**load BFR squats than during high**-**load squats, together with a reduction in subsequent VJH. This discrepancy may be explained by differences in the CT**-**BFR protocol, including exercise volume, load intensity, cuff pressure, and recovery time. In particular, the high**-**repetition protocol used by Cleary and Cook may have produced greater fatigue, which could have limited both muscle activation and subsequent jump performance. In the present study, the control condition of traditional high**-**load CT control condition may also have been strong enough to induce PAPE. This may have reduced the magnitude of between**-**condition differences in RMS, MF, and CMJ performance.

The MF results should be viewed as fatigue related information rather than direct evidence of potentiation. MF reflects the frequency characteristics of the EMG signal. A decrease or unstable change in MF may indicate fatigue**-**related changes in the working muscles. In the present study, the MF responses suggest that increased muscle activation and fatigue**-**related changes may have occurred at the same time after CT**-**BFR. This is important because higher pressure can increase metabolic stress and perceptual strain. Therefore, the CMJ response should be interpreted together with RMS, BLa, and performance outcomes.

BLa provided further evidence of metabolic stress after CT**-**BFR. During BFR exercise, reduced oxygen availability and restricted venous return may increase glycolytic contribution and lactate accumulation (Pearson and Hussain, 2015; Kim et al., 2018). In the present study, BLa increased significantly after the intervention, indicating that CT**-**BFR induced a clear metabolic response. The higher BLa response under 70% AOP than under 60% AOP suggests that 70% AOP provided a stronger metabolic stimulus. In contrast, 60% AOP may have allowed relatively greater oxygen delivery and lactate clearance, which may explain its weaker PAPE response. This interpretation is consistent with Suga et al. (2010), who reported that partial venous occlusion at 60% AOP during low**-**load squatting allowed sufficient oxygen delivery and reduced glycolytic flux and lactate production.

However, a higher pressure did not lead to better performance (Wernbom and Aagaard, 2020; Lauber et al., 2021). Under 80% AOP, the combination of higher cuff pressure and the CT-BFR protocol may have increased fatigue, which could have limited the expression of PAPE (Wernbom and Aagaard, 2020; Lauber et al., 2021). Higher cuff pressure can further restrict local blood flow, reduce oxygen availability, and slow the removal of metabolites. As a result, metabolic by**-**products such as H^+^, inorganic phosphate, and BLa may accumulate in the working muscles. These changes can disturb excitation**-**contraction coupling and reduce the efficiency of force production (Scott et al., 2016). Excessive metabolic stress may also increase inhibitory afferent feedback from the working muscles (Wernbom et al., 2008; Kennedy et al., 2015). This could limit central motor drive during the subsequent CMJ. Therefore, the higher**-**pressure condition may have shifted the acute response from potentiation to fatigue. This may explain why performance did not improve significantly during recovery.

Overall, the PAPE response after CT**-**BFR is likely affected by several factors, including load intensity, exercise mode, cuff width, individual AOP, and whether the exercise is performed to failure (Murray et al., 2021). The 70% AOP condition appeared to provide a more favorable balance between neuromuscular stimulation and fatigue, which may partly explain the superior PAPE responses observed under this condition.

## 5 Limitations

Several limitations should be acknowledged. First, the present study examined only vertical jump performance, and the findings may not be directly transferred to other sport**-**specific tasks, such as sprinting, change of direction, or repeated explosive actions. Second, the relatively small sample size and inclusion of only young trained males may have limited the statistical power and generalizability of our findings to other populations, particularly clinical populations. Previous evidence has shown that BFR combined with exercise can improve lower-limb function in individuals with hemiplegia (Feng et al., 2025). However, the applicability of CT-BFR in rehabilitation settings requires further investigation. Third, the external load and exercise volume were not matched between the traditional CT and CT-BFR conditions, which should be considered when interpreting the effects of BFR and cuff pressure. Fourth, repeated maximal CMJ assessments during recovery may have introduced a small additional neuromuscular demand, which could have contributed to fatigue accumulation and should be considered when interpreting the temporal profile of PAPE responses. Finally, the study did not directly measure muscle oxygenation, central fatigue, peripheral fatigue, or molecular signaling responses. Therefore, the proposed mechanisms should be interpreted as indirect explanations based on performance, sEMG, and BLa responses.

## 6 Conclusion

The present study showed that CT**-**BFR produced cuff pressure**-**specific acute responses in CMJ performance, sEMG activity, and BLa. Among the tested pressures, 70% AOP showed the most consistent acute response pattern under the present experimental conditions, especially during the 8–12 min recovery window. This condition improved CMJ performance and increased normalized RMS in selected thigh muscles, while producing a clear metabolic response without the performance decrement observed under 80% AOP.

These findings suggest that 70% AOP may represent a potentially effective pressure level for inducing acute lower-limb PAPE under similar experimental conditions. However, further research is needed to confirm whether these acute responses translate into long-term adaptations.

## Data Availability

All relevant data used in this study are available upon reasonable request to the corresponding author. Requests to access the datasets should be directed to XW, 13792111077@163.com.
